# Comparing Data Collection Tools for Zoo Management Decision-Making: A Case Study Examining Behavioral Measures of Humboldt Penguin Bond Strength

**DOI:** 10.3390/ani12213031

**Published:** 2022-11-03

**Authors:** Julia Galante, Susan W. Margulis

**Affiliations:** 1 Department of Animal Behavior, Ecology, and Conservation, Canisius College, Buffalo, NY 14208, USA; 2 Department of Biology, Canisius College, Buffalo, NY 14208, USA

**Keywords:** Humboldt penguin, activity budget, Zoo Monitor, Animal Behaviour Pro, exhibit use, pair-bonding

## Abstract

**Simple Summary:**

Systematic data collection has become an important practice in zoos. Here, we describe the results of a two-year study on exhibit use and pair-bonding in a colony of Humboldt penguins. We further compared two different data collection apps to evaluate their effectiveness in and suitability for evaluating pair-bond strength. There was considerable individual variation in penguin behavior in terms of activity, time spent in water, and courtship behaviors. The longer pairs had been bonded, the more time they spent in close proximity. We note the importance of evaluating data collection tools before embarking on a study to ensure that the tool will provide data in a form that can be easily interpreted.

**Abstract:**

Systematic data collection has become increasingly important in zoos as it facilitates evidence-based decision-making. Here, we describe the results of a two-year study on exhibit use and pair-bonding in a colony of Humboldt penguins. We used two different data collection apps to evaluate their effectiveness and suitability for evaluating pair-bond strength. Data were collected using instantaneous scan sampling and all-occurrence sampling 2–3 times per week for 2 years for a total of nearly 240 h of observation (19 h with one system and 219 h with the other system). The activity patterns (in particular, time spent in the water) differed amongst penguins and between the two data collection tools. Patterns of courtship-related behaviors varied tremendously across individuals. The longer pairs had been bonded, the more time they spent in close proximity. We highlight two important considerations for institutions aiming to collect such systematic data. First, it is critical to interpret all findings in context by incorporating husbandry details and keeper insights to highlight explanations that may not be readily apparent from the data. Second, one must explore all aspects of any data collection system before committing to its use—system setup, ease of data collection, format and accessibility of exported data. Not doing so may negate the value of systematic data collection by limiting the use and interpretability of the data.

## 1. Introduction

Behavioral observations have long been a staple of zoo animal management. Whether anecdotal reports by keepers, observations by students, volunteers, or research staff, or formal academic studies, behavioral research is arguably the most common type of research conducted in zoos. Several surveys over the years have identified behavior as the single largest area of research conducted in North American zoos [1], and similar findings have been reported for British and Irish Association of Zoos and Aquariums (BIAZA) zoos [2]. The importance of behavioral information, both anecdotal and systematic, for making management and husbandry decisions has been noted in recent years [3,4,5,6], with tools such as WelfareTrak^®^ (www.welfaretrak.org (accessed on 1 September 2022)) facilitating such efforts.

Many automated tools for behavioral data collection have been developed in the past 10–15 years, becoming more affordable and more available to zoo researchers and animal care staff. The advent of such tools, available at little or no cost, has enhanced the efficiency and consistency of such studies [7,8]. A growing emphasis on zoo animal welfare—particularly indicators of positive welfare [9]—has led to a broader and more comprehensive approach to incorporating the results of both subjective and objective observations into zoo decision-making and evidence-based management.

Given the growing importance of behavioral research in zoos to inform management decisions, and the increasing diversity and availability of data collection tools, understanding the ways in which different data collection systems may be used depending on the nature of the research or management question has become an increasingly complex decision. Some data collection tools phase out over time as support for their maintenance ebbs and flows. Other tools may be designed for one purpose but find use in other realms as well. The advantages, disadvantages, and appropriateness of each app or tool must be carefully assessed for each research or management application so that the most appropriate system can be selected for the specific situation.

The Humboldt penguin (*Spheniscus humboldti*) is exhibited in many zoos and aquariums throughout North America. Named after the Humboldt Current, the cold current that flows northward from Antarctica, the species can naturally be found on the western coasts of Chile and Peru. The Humboldt penguin is currently listed as vulnerable by the IUCN (International Union for the Conservation of Nature) due to wild populations declining as a result of climate change and human disturbance. These factors are due to the remoteness of colonies which are mainly restricted to islands and inaccessible sea caves (43° S to 5° S) [10]. The species is considered to be monogamous and shows no sexual dimorphism—they may be sexed through the use of a proctoscope or by observing their behavior during copulation [3]. Wild mated pairs will construct a nest out of their own guano, where they raise their offspring. Since the mid-19th century, guano has been commercially exploited as a fertilizer and guano deposits were removed down to the bare rock, making it impossible for the birds to excavate burrows [11,12].

There are many different definitions of pair bonds across taxa. Pair bonds are characterized by a suite of behaviors that differ amongst species and involve a relationship between a single male and a single female [13]. Oftentimes, this assumes a long-term relationship beyond a single breeding event. Amongst avian species, pair-bonded individuals express unique behaviors, such as allo-preening, duetting and courtship displays. A high degree of proximity maintenance accompanies a pair bond in several definitions and some even argue that the amount of time a pair spends in proximity is indicative of the pair bond’s strength [14].

Although threatened in the wild, the Humboldt penguin is one of the most popular penguin species found under managed care. Maintaining a sustainable population in zoos is critical and has played an important role in conservation efforts for this species. Despite their popularity, they remain understudied. There are currently 424 Humboldt penguins housed in 21 separate facilities that are accredited by the Association of Zoos and Aquariums (AZA) [15], which makes this species the second largest population in North American zoos, second only to the African penguin (*Spheniscus demersus*) [16]. Humboldt penguins in AZA accredited facilities are part of the Species Survival Plan (SSP), a breeding program that ensures genetic diversity and the sustainability of the managed population. Thus monitoring reproductive behavior, bond strength, and pair affiliation may serve as good indicators of likely breeding success.

Here, we discuss the results of a 2-year study on behavior and exhibit use in a colony of Humboldt penguins. Our aims were (1) to examine behavior as an indicator of pair-bond strength amongst breeding pairs; (2) to evaluate exhibit use in the colony; and (3) to offer recommendations of different data collection apps based on the nature of the data required to answer specific questions. We predicted that pairs that were more often together would exhibit more frequent affiliative and reproductive behaviors as indicators of positive welfare. If behavioral indicators can be used to predict bond strength and welfare, findings such as these could be used to inform breeding recommendations and management decisions.

## 2. Materials and Methods

### 2.1. Study Site and Subjects

We observed a colony of 15 Humboldt penguins at the Aquarium of Niagara, in Niagara Falls NY, USA. The colony contained six mated pairs, two juveniles, and one unmated adult male. The penguin exhibit at the Aquarium of Niagara opened in 2018 and was designed to hold a maximum of 24 birds. The exhibit measures 13 m by 7 m with a total volume of 550 cubic meters including a 57,000 L pool with a depth of 1.2 m. All penguins were banded (males on the right wing, females on the left). Mated pairs had the same-colored bands (Table 1).

### 2.2. Procedures

**Figure 1:**
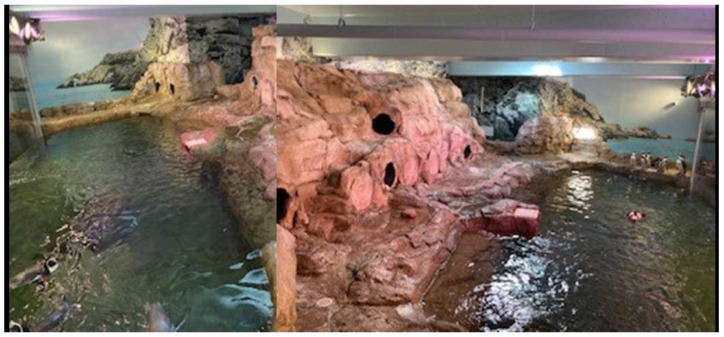
Penguin exhibit “map” for noting penguin location in Zoo Monitor. The shape of the exhibit necessitated using 2 photos and combining them. We adjusted for the angle discrepancy in calculations.

We collected data 2–3 times per week. Following a protocol from a previous study [17], we used Animal Behaviour Pro^®^ from August to October 2020, for data collection (the breeding season ended in August 2020). Animal Behaviour Pro^®^ is an inexpensive app designed for behavioral sampling by permitting the user to set up an ethogram with modifiers. The original study used this tool to monitor basic activity patterns and behavior changes following the move to the new penguin exhibit using instantaneous scan sampling [18] (Animal Behaviour Pro ethogram; Table 2). Behaviors related to pair-bonding were tabulated as all-occurrence behaviors. Observations with Animal Behaviour Pro occurred for 10 min blocks with a scan every minute. During each day of observation, we collected six 10-min sets of data (60 min in total), with a short (3–5 min) break between each 10-min observation. Thus, we collected 60 data points per penguin over 60 min of data collection during each day of observation. In October, 2020 we switched our data collection to ZooMonitor^®^ which allowed us to better track location within the exhibit. ZooMonitor allows the user to collect data on states, all-occurrence behaviors, and exact locations within the exhibit. Given the range of behaviors observed in the penguins, we decided that location data would be more informative than activity. If penguins were in the water, they were swimming; penguins on the land were either standing or lying down and this difference was not relevant to our research. We expanded on the number of all-occurrence behaviors recorded to better evaluate pair bond strength. Given the amount of information we were collecting on each scan, we found it necessary to increase the interval between scans from 1 min to 1.5 min. This increased the duration of an observation from ten minutes to 15 min. We continued to collect 60 point observations on each penguin during each day of data collection. The total observation time per observation day now increased from 1 h to 1.5 h. The ethograms differed somewhat for the two systems: with Animal Behaviour Pro, we collected activity budget data (Animal Behaviour Pro ethogram; Table 2). When we switched to Zoo Monitor, we recorded the location of each individual bird at each point scan on a visual representation of the exhibit (Figure 1). We collected all-occurrence behavioral observations on a small subset of behaviors related to pair-bonding (Zoo Monitor ethogram; Table 3). We opted not to collect activity budget data, as these data were not useful to our key questions. Data collection ended in March 2022. Zoo Monitor data encompassed all of the 2021 breeding season and half of the 2022 breeding season.

### 2.3. Data Analysis

For Animal Behaviour Pro data, we calculated activity budgets based on the percent time spent in each state behavior during each day of observation [19,20,21]. We tabulated the frequency of Animal Behaviour Pro behaviors related to pair-bonding (aggression, copulation, and allo-preening). We did this also for the Zoo Monitor all-occurrence data. We calculated the average proximity of pairs from the Zoo Monitor data by first removing observations in which both members of the pair were in the water (because of their constant movement when swimming, intra-pair proximity is approximate at best when both birds were in the water simultaneously). We used the remaining data to calculate average proximity of pairs by first converting pixels to meters based on exhibit dimensions, then averaging this across all points in which one or no members of a pair were in the water [20,22,23]. All statistical analyses were conducted using Excel and R for data tabulation, visualization, and analysis. Activity budget data from Animal Behaviour Pro were analyzed using a Kruskal Wallis test with a post hoc Dunn’s test [24,25]. We used Chi square tests to examine frequencies of all-occurrence behaviors in order to look for differences amongst pairs. We had 11 times more data from Zoo Monitor observations (219 h) than Animal Behaviour Pro observations (19 h), due to the differing scan intervals (scans every 1.5 min versus scans every minute) and months of data collection. To account for this difference when analyzing these behaviors, we adjusted the Zoo Monitor all-occurrence behavior data downwards thus giving us comparable amounts of observation time. We only did this for the all-occurrence behaviors, since all other behaviors were tabulated based on percent of total observation time. To compare Zoo Monitor intra-pair proximity (that is, how far apart pair members were from one another), we averaged the intra-pair proximity by day across the entire 17-month dataset for a mean intra-pair proximity measure. We used a Kruskal-Wallis test with a Dunn’s post-hoc test [24,25] to identify differences in proximity amongst penguin pairs, and a Levene’s test for homogeneity of variances amongst pairs [26].

## 3. Results

All analyses are based on 19 days of data using Animal Behaviour Pro (19 h of data) and 146 days of data using Zoo Monitor (219 h of data). All penguins were observed simultaneously. We were unable to collect data during the period when the Aquarium was closed due to COVID restrictions (March 2020–July 2020).

### 3.1. Activity Budgets

**Figure 2:**
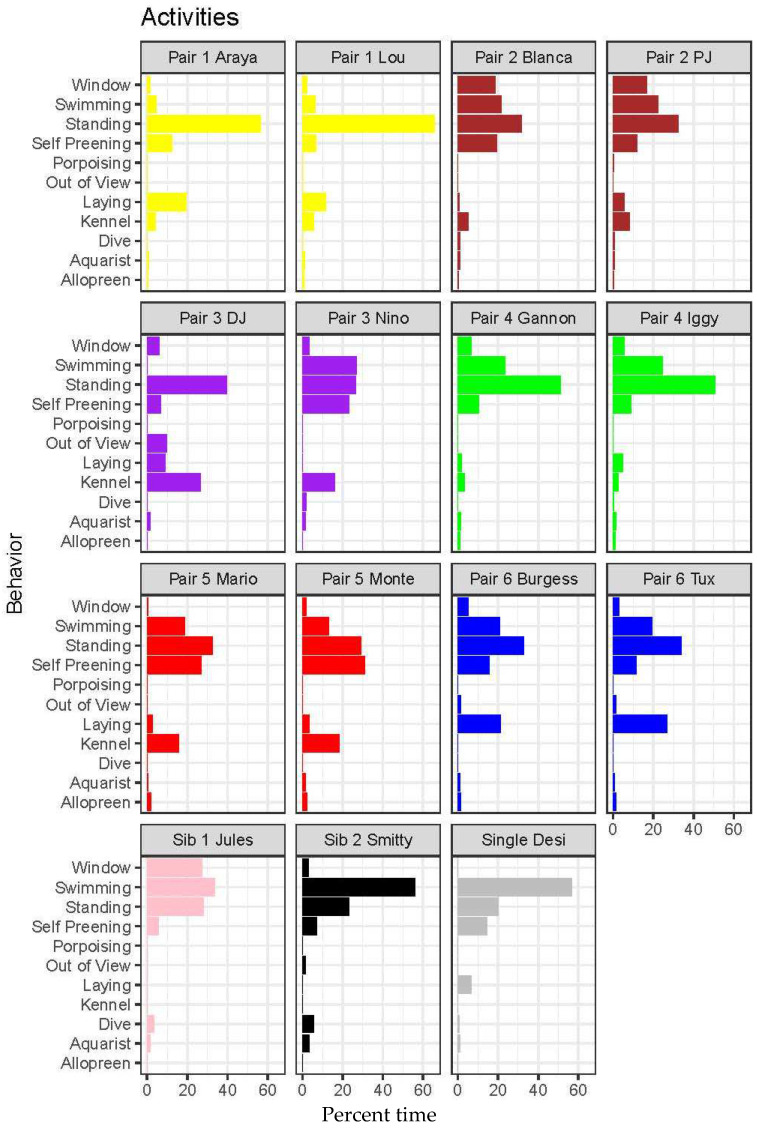
Activity budgets of penguins based on data collected with Animal Behaviour Pro. The definitions of each behavior may be found in Table 2.

Activity budget data from Animal Behaviour Pro were analyzed for the six mated pairs as well as the juvenile siblings and the single individual. The activity budgets that were recorded varied amongst individuals (Figure 2). Kruskal Wallis and pairwise *t*-tests were used to analyze three activities—Swimming (Kruskal-Wallis Χ^2^ = 104.69, df = 14, *p* < 0.0001), Kennel (Χ^2^ = 53.607, df = 14, *p* < 0.0001) and Standing (Χ^2^ = 70.566, df = 14, *p* < 0.0001). Dunn’s post-hoc tests with Bonferroni corrections revealed that these patterns were largely driven by a small number of penguins. For swimming, the unpaired male “Desi” and the young male “Smitty” spent significantly more time swimming than any other individuals. Additionally, the oldest female in the colony, “DJ” spent significantly less time swimming than most of the other penguins (detailed statistical output is available in the supplemental materials, Table S1). For kennel (i.e., time spent in nest burrow), the difference was driven largely by several individuals who did not use a kennel/nest burrow: the blue pair “Tux” and “Burgess”, and the young siblings “Jules” and “Smitty”. Finally, the pattern for standing was driven largely by the yellow pair “Araya” (female) and “Lou” (male) who spent significantly more time standing than did any other individuals.

**Figure 3:**
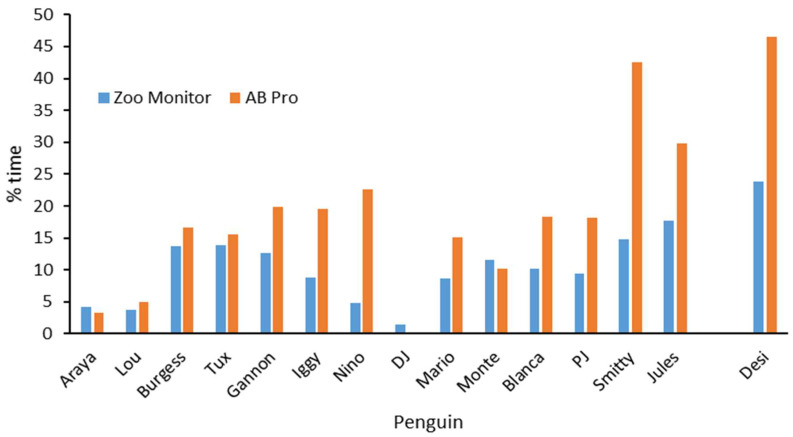
Percent time spent in water using two different data collection apps Animal Behaviour Pro data were collected from August to October 2020; Zoo Monitor data were collected from October 2020 to March 2022.

To calculate the amount of time birds spent in the water using the different data collection systems, we used the percent time in which individuals were scored as “swimming” from Animal Behaviour Pro, and the percent of scans in which penguins were identified as being in the water using Zoo Monitor. The amount of time birds spent in the water differed between the two data collection applications. (Figure 3). Penguins were measured as spending significantly more time in the water using Animal Behaviour Pro than Zoo Monitor (t = 3.79, df = 14, *p* < 0.002). Approximately 2/3 of the Animal Behaviour Pro data were collected outside of the breeding season, while approximately half of the Zoo Monitor data were collected during the breeding season. Similar to the Animal Behaviour Pro activity budget patterns, “Desi” and “Smitty” (along with “Jules”, the other juvenile) spent notably more time in the water than did other birds.

### 3.2. Pair-Bonding Behaviors

**Figure 4:**
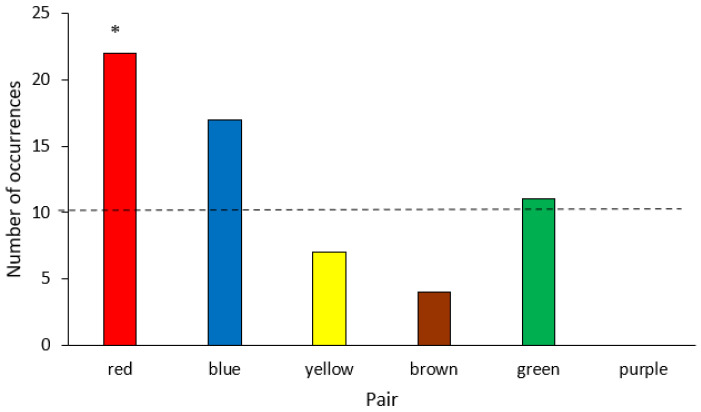
Frequency of allo-preening over 19 observation days based on Animal Behaviour Pro data. Red pair allo-preened significantly more than all other pairs, and Purple pair did not allopreen at all. Dashed line equals the expected frequency of allo-preening if there were no differences amongst pairs; * = *p* < 0.05.

Only the six mated pairs were included in this analysis. Duetting was not tabulated using Animal Behaviour Pro; copulation occurred too rarely to analyze. There was sufficient data to evaluate frequency of allo-preening. There were significant differences in the frequency of allo-preening by pair (Χ^2^ = 33.9, df = 5, *p* < 0.001), driven largely by the Red pair allo-preening significantly more than other pairs, (Χ^2^ = 14.4, df = 5, *p* < 0.005) and the Purple pair not allo-preening at all (Figure 4).

**Figure 5:**
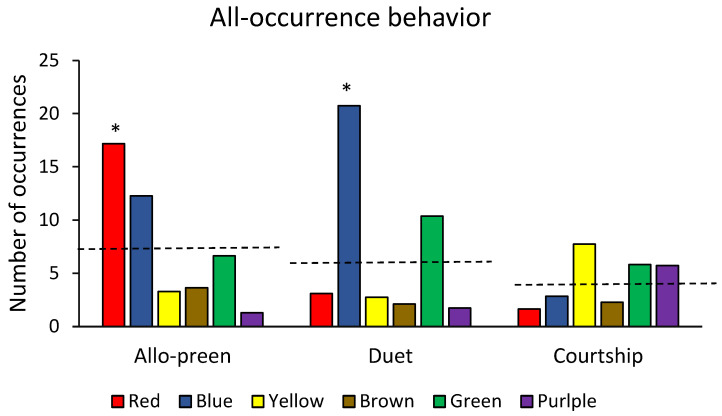
Frequency of pair-bonding behaviors across 6 mated pairs of Humboldt penguins based on 219 h of Zoo Monitor data. Frequencies were adjusted downwards by a factor of 11 to account for the differences in observation time across the two data collection systems. Dashed lines indicate the expected frequency of behaviors assuming no differences amongst pairs. * = *p* < 0.05. See text for details.

There was sufficient Zoo Monitor data to examine all three pair-bond related behaviors (courtship, duetting, and allo-preening). Two of three behaviors showed significant differences in frequency across pairs (allo-preen: Χ^2^ = 25.5, df = 5, *p* < 0.001; duetting: Χ^2^ = 41.9, df = 5, *p* < 0.001; courtship: Χ^2^ = 6.8, df = 5, N.S Figure 5). The Red pair showed significantly more allo-preening than expected (Χ^2^ = 12.93, df = 5, *p* < 0.05), and the Blue pair showed significantly more duetting (Χ^2^ = 28.52, df = 5, *p* < 0.001). Both Animal Behaviour Pro and Zoo Monitor revealed similar patterns for allo-preening (the only comparable behavior from the two datasets). The results did not differ between these two periods for allo-preening (Χ^2^ = 2.54, df = 5, NS).

### 3.3. Proximity

**Figure 6:**
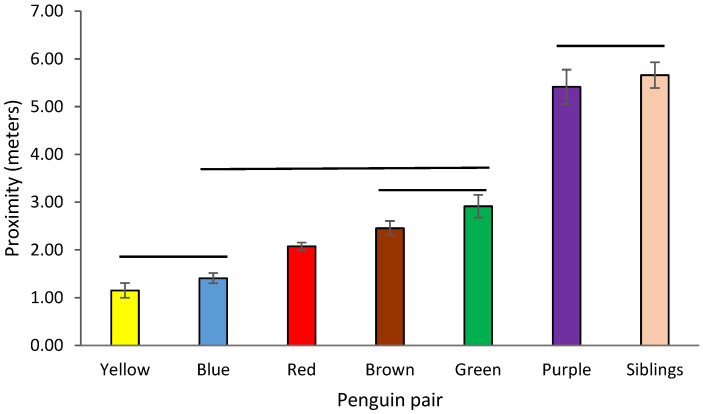
Intra-pair proximity amongst penguin pairs based on Zoo Monitor data. Lines indicate pairs that did not differ significantly from one another. Any bars not connected by lines are significantly different (*p* < 0.05). See supplemental materials for details statistical output.

Intra-pair proximity was calculated with Zoo Monitor data only. Pairs differed significantly in mean intra-pair proximity (Kruskal-Wallis test, Χ^2^ = 61.68, df = 6, *p* < 0.0001). A Dunn’s post-hoc test with Bonferroni correction showed that all pairwise comparisons were significantly different with the following exceptions: Purple and Siblings were not significantly different, nor were Blue and Yellow nor Brown and Green. Red did not differ significantly from Blue, Brown and Green (Figure 6; statistical output available in supplemental materials; Table S2). Variance in intra-pair proximity differed significantly across pairs (Levene’s test, *F* = 54.81, df = 6, *p* < 0.0001). The same pairings that did not differ in mean proximity did not differ in variance of proximity based on a Dunn’s post-hoc test. The only additional pairing that did not differ, was the Brown and Green comparison.

**Figure 7:**
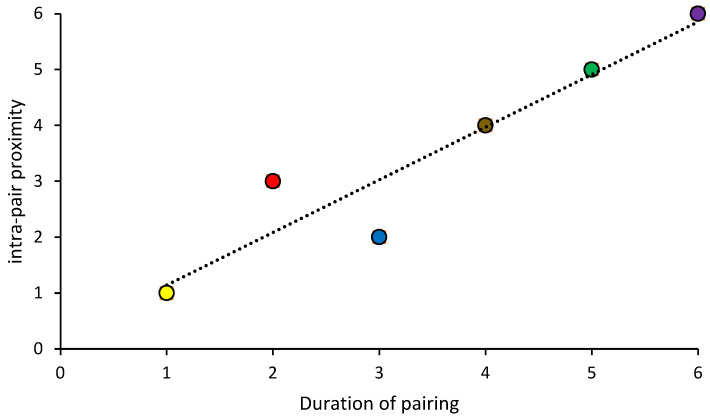
Relationships between duration of pairing and intra-pair proximity based on Zoo Monitor data. For duration of pairing, 1 = together the longest, 6 = most recently established pair. The longer pairs have been together, the more time they spend with one another (Spearman Rho rank correlation, 0.94).

Intra-pair proximity was strongly correlated with the length of time pairs had been together (Spearman Rho rank correlation = 0.94; Figure 7). The longest established pair in this colony, the Yellow Pair, have been bonded since 2005 (Table 1). They have the smallest intra-pair proximity, indicating they spend much of their time together. The Purple Pair was the most recently established, and this pair had the greatest average proximity.

**Figure 8:**
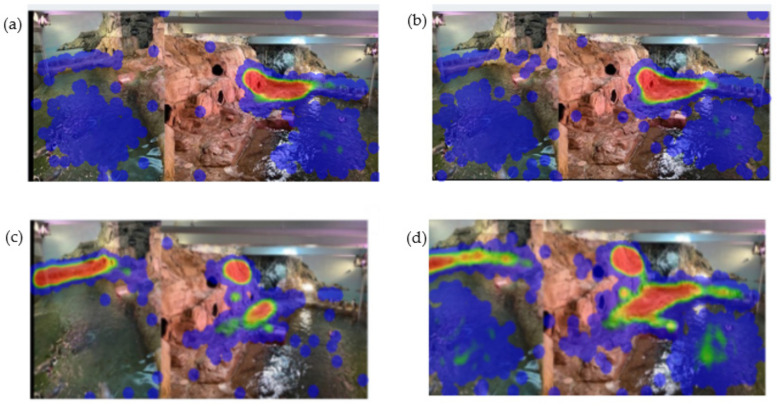
Heat maps generated by Zoo Monitor. Red indicates areas in which individuals spent the most time, and blue the least time. Upper panels: Yellow pair (female “Araya” (**a**) on the left, male “Lou” (**b**) on the right). Lower panels: Purple pair (Female “DJ” (**c**) on the left, male “Niño” (**d**) on the right), Heat maps for all remaining penguins are available in the supplemental material (Figures S1–S6).

**Figure 9:**
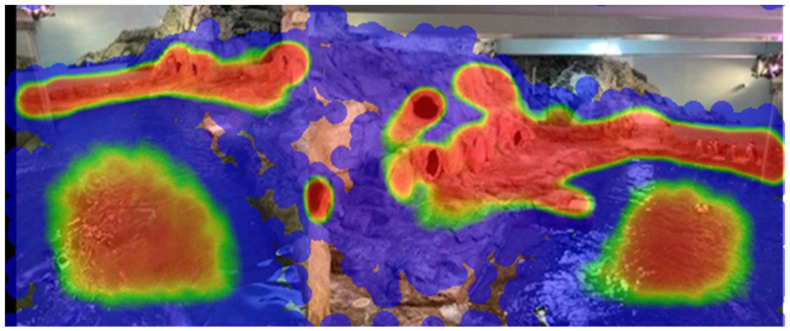
Combined heat map for all penguins over 146 observation days. Areas of intense use are shown in red; areas that were rarely used are shown in blue.

Zoo Monitor-generated heat maps confirm the patterns above. The heat maps show where the penguins spent the most time. The red highlights areas in which an individual spent the most amount of time, while the blue areas are used the least. The distance data suggest that “Araya” and “Lou” (Pair 1—Yellow Pair) spent the most time together, with their average distance apart being 1.2 m. “DJ” and “Niño” (Pair 3, or Purple Pair—the most recently- established pair) spent the least amount of time together out of all the pairs, with their average distance apart being 5.12 m. Lou and Araya’s heat maps suggest that they spent most of their time in the same areas (a single area of intense use), while DJ and Nino’s heat maps suggest that this pair spent most of their time in different areas based on the several areas of intensive use (Figure 8). A combined heat map for all penguins provides a visual representation of overall exhibit use patterns (Figure 9).

## 4. Discussion

### 4.1. Bond Strength and Intra-Pair Proximity

Intra-pair proximity has previously been suggested as an indicator of bond strength. We found that the best predictor of intra-pair proximity was duration of pairing. The longer pairs have been bonded, the more likely they were to be in close proximity. Behaviors associated with pair-bond strength, including courtship, duetting, and allo-preening [13], by definition, occur when pair members are close together.

Overall, our findings suggest that penguin behavior and proximity need to be interpreted in context in order to more accurately interpret the results. While we found significant differences amongst pairs in some behaviors indicative of bond strength, patterns were inconsistent and not always related to intra-pair proximity, which proved to be the best indicator. Looking solely at the data without details from animal care staff can lead to misleading conclusions. For example, the Brown Pair showed the least number of all-occurrence behaviors related to pair-bonding and bond maintenance, yet this pair incubated eggs (Table 1). Several additional pairs incubated dummy eggs (that is, they were not recommended to breed), leading to extended time in kennels and a reduction in courtship-related behaviors but these pairs would be considered well-bonded. The Blue Pair chose not to occupy a nest box but preferred to defend a small territory on the rocky beach area. Consequently, they were more likely to be visible to observers.

All-occurrence pair-bonding behaviors vary between each pair and may depend on factors such as age, time since pairing, and whether the pair-bond was forced or occurred naturally (a forced pairing is made by placing a male and female together in a separate area until animal care staff confirm likely establishment of a bond based on behavior). For example, the Green Pair was forced to pair by animal care staff in May 2020, but were also the most recent pair to exhibit nesting behaviors and receive a dummy egg to incubate. This supports the idea that establishment of a strong pair bond takes time, and pairs that have been together longer demonstrate stronger pair bonds based on proximity maintenance.

### 4.2. Activity Budgets and Exhibit Use

Overall, activity budget data provided limited information regarding pair bond strength and positive welfare state. The most meaningful finding from activity budget data was individual differences in swimming. Based on both data collection methods, unpaired males (the same two individuals throughout both data collection periods) spent significantly more time in the water than did paired males. Previous research [15] indicated that all birds spent more time in the water in their new exhibit, which is not surprising, given the much larger pool and larger exhibit size. A recent study on Humboldt penguins [27] found that on average, penguins spent less than 20% of their time swimming. Penguins are known to reduce their time spent in the water during the breeding season (9.1%) [28]. When breeding seasonality is considered, the difference between our two data collection periods makes sense, given that much of the Animal Behaviour Pro data was collected outside of the breeding season. Other factors may also influence the amount of time spent in the water. The addition of enrichment has been found to increase the time penguins spend swimming [27], and although we were not collecting data on this, we anecdotally noted when animal care staff added enrichment during and observation. This likely had a similar impact on time spent swimming as some of the birds would enter the water to interact with enrichment. While informative, this activity budget data was not helpful in identifying exhibit use patterns of interest and provided limited data on bond strength. Adjusting the ethogram might have clarified these issues to some extent, but ultimately percent time spent swimming and percent time in the water provide the same information; we suggest that exhibit use/location within the exhibit was more informative for our needs than was activity budget.

The heat maps generated by Zoo Monitor provide a valuable visual indication of the amount of time pairs spend in the same area and thus in close proximity to one another. Analyzing these data to calculate mean distance between pairs was not trivial and may be beyond what is reasonable to expect from zoo staff without substantial statistical knowledge and expertise with R or similar programming software. However, our results suggest that intra-pair proximity may be a good indicator of bond strength and positive welfare. Although we cannot directly correlate this to reproductive success, it may provide a simple measure to help gauge likely breeding success.

### 4.3. Comparing Data Collection Tools

All data collection tools and methods have advantages and disadvantages [4,7,8,29,30]. The information that is necessary to set up a data collection system, the extent to which staff can analyze and interpret data, the cost of apps, and the degree of app support all must be taken into consideration. When we took over this study from its originators [17], we opted to maintain consistent data collection. Thus, we used Animal Behaviour Pro with an emphasis on activity. We realized however, now that the penguins had been in their exhibit for two years, other questions were of greater interest and could be better answered with a different data collection method. While altering the Animal Behaviour Pro ethogram or adding additional channels for data collection (for example, location or all-occurrence data) might have been useful, we had some challenges working with the Animal Behaviour Pro app. The app would sometimes crash unexpectedly leading to data loss, updates and responses to problems were not always received in a timely manner, and the format of the Animal Behaviour Pro data output was more challenging to interpret, with each observation saved as a separate data file. Animal Behaviour Pro allows for parallel but mutually exclusive channels of behaviors to be collected (for example, activity and location) which can be useful in some situations. Zoo Monitor’s ability to add a map or image and place animals there is a strength; Zoo Monitor technical support is readily available and the app is updated regularly; data may be downloaded as a single csv file for any period of time during which data have been collected. This greatly facilitated our ability to analyze the entire dataset, or specific subsets, with relative ease. For animal management purposes, the heat maps provide an easily interpreted visual representation of exhibit use, however the current version does not permit easy analysis of this information. If a zoo was interested only in overall exhibit use patterns, a combined heat map can be very informative (Figure 9). Of course, certain additional features could increase its usefulness further. Ideally, being able to clearly define specific areas on an exhibit map or image could greatly facilitate analyses. For example, delineating the pixels that comprise a nest box, a feeding area, a keeper access door, or other key exhibit features such as the pool in this study, would have greatly simplified data analysis. If we were able to do this, we might have been better able to determine when pair members were in the same place at the same time. Since we had limited data from Animal Behaviour Pro for this investigation (19 days, compared to 146 days with Zoo Monitor), direct comparisons were not always possible. We note that both systems have strengths and weaknesses, and both are more useful than anecdotal notes and observations. Animal Behaviour Pro costs very little ($0.99 US); Zoo Monitor is free to AZA-accredited institutions and $50 US a year to unaffiliated researchers. This difference in cost may explain the enhanced technical support available for Zoo Monitor.

We have suggested recommendations for institutions aiming to utilize data collection tools for evidence-based management purposes. We encourage institutions to explore all readily available tools and evaluate: (1) ease of set up; (2) ease of use; (3) output format; and (4) availability of technical support, before committing to a particular app. Output format is often overlooked when a project is conducted yet this can lead to significant complications and problems when data analysis and interpretation is initiated.

## 5. Conclusions

In this study, we found that the duration of pairing was the best predictor of intra-pair proximity in this Humboldt penguin colony. It is important to evaluate data collection tools to identify the system that will provide the most relevant and easy to use information. Here, we found that activity and exhibit use provided comparable information (i.e., swimming and in water are equivalent), yet different tools were appropriate for quantifying each of these. The dizzying array of available apps and tools range from free to many thousands of dollars [7], and programmers can often tailor a tool to very specific studies. However, in the scope of zoo-based research, with an aim towards broad applicability, a tool such as Zoo Monitor is likely to facilitate cross-institution comparisons more readily than customizable tools.

When it comes to data collection, no single, ideal tool will be appropriate in all situations. In this study, the appropriate tool changed as the focus of our investigation shifted. Although this can limit the ability to make long-term comparisons, ultimately, switching to a more appropriate app can provide more accessible and useful information for both research and management purposes.

## Figures and Tables

**Table 1:** Subjects of study. Studbook numbers follow the house names of all subjects. * = forced pairing. All pairs incubated dummy eggs with the exception of the “Brown”-banded pair. Dummy eggs are given to pairs who show nesting behavior but are not recommended to breed. Incubation periods span the length of the breeding season (approximately mid-February through mid-August).

Male	Female	Band Color	Date of Pair Bonding	Incubation Periods
Mario (944)	Montana (962)	Red	April 2007	17 March–25 April 2021; 23 February 2022–end of data collection
Tux (676)	Burgess (859)	Blue	November 2015	
Lou (828)	Araya (655)	Yellow	April 2005	15 March–27 April 2021 22 May–3 July 2021 7 August–20 August 2021 10 February–13 March 2022
PJ (1205)	Blanca (1288)	Brown	February 2017	14 February–29 March 2021 7 May–19 June 2021 20 February 2022–end of data collection (eggs)
Iggy (1263)	Gannon (1314)	Green	May 2020 *	1 March 2022–end of data collection
Niño (1341)	DJ (505)	Purple	May 2020	12 April–21 May 2021
Smitty (1491)	Jules (1490)	Siblings	N/A	17 February 2022–end of data collection

**Table 2:** Animal Behaviour Pro Ethogram. All behaviors were considered to be states, and the state of each individual penguin was recorded every minute during each 10-min observation period. Aggression, copulatory behavior, and allo-preening were analyzed as all-occurrence behaviors to support comparisons with Zoo Monitor data.

Behavior	Definition
Aggression	Subject is engaged in an agonistic interaction with another penguin (initiating or receiving) including pecking, vocalizing in close proximity, charging, chasing, territorial display etc.
Copulatory behaviors	Subjects will stack vertically on top of each other and flap wings, fertilization is internal.
Interacting with Aquarist	Receiving fish, receiving care or preening, or within a close proximity to Aquarium staff.
Kennel	Subject is inside of a nest box.
Laying	Subject is on the ground of the exhibit with the keel touching the floor.
Standing	Subject is on the ground of the exhibit with keel off of the ground
Swimming	Subject is in the pool on the surface with head and beak still out of the water
Porpoising	Subject is using the pool and jumps all the way out of the water. Usually accompanied by bursts of speed underwater.
Dive swimming	Subject’s whole body is submerged in the pool.
Self-Preening	Subject is using beak to groom feathers of themselves
Allopreening	Subject is using beak to groom feathers of another bird. Use the receiver function
At window	Within 0.2 m of exhibit glass on both panels on the sides of the pool.
Out of view	Subject has been removed from exhibit by a keeper.

**Table 3:** Ethogram for Zoo Monitor. Behaviors recorded whenever they occurred during each 15-min observation period. In addition, the location of each penguin was noted on the map (Figure 1) every 1.5 min during each 15-min observation period.

Behavior	Definition
Allo-preen	One bird uses beak to groom feathers of another
Duet	Pair vocalize together
Courtship	Mount or attempted mount
Keeper enters	Keepers comes into exhibit, usually to feed or enrich
Keeper leaves	Keeper exits exhibit
Aggression	Penguin pecks or chases another

## Data Availability

Data and R Code for this study are available from the corresponding author upon reasonable request.

## Supplementary Materials

The following supporting information can be downloaded at: https://www.mdpi.com/article/10.3390/ani12213031/s1, Table S1: Results of Dunn’s post-hoc tests for activity budgets; Table S2: Results of Dunn’s post-hoc tests for proximity; Figure S1: Heat maps of “Blue Pair”; Figure S2: Heat maps of “Red Pair”; Figure S3: Heat maps of “Green Pair”; Figure S4: Heat maps of “Brown Pair”; Figure S5: Heat maps of siblings; Figure S6: Heat map of unpaired male.
