# Supplementary material for: Comparing Data Collection Tools for Zoo Management Decision-Making: A Case Study Examining Behavioral Measures of Humboldt Penguin Bond Strength
Source: Animals (Basel). 2022 Nov 3;12(21):3031. doi: 10.3390/ani12213031 (PMC9657121; doi:10.3390/ani12213031)
Supplement: Supplementary file 1 [file animals-12-03031-s001.zip › animals-1928176-supplementary.pdf]

Supplemental Table S1. Results of Dunn's post-hoc tests for activity budgets (Animal Behaviour Pro data). Table includes Z scores and adjusted P values (significant differences highlighted in yellow). See Section 3.1.

	SWIM		KENNEL		STAND	
Comparison	Z	P.adj	Z	P.adj	Z	P.adj
Araya - Blanca	-2.5605	1.000	-0.7218	1.000	3.00311012	0.281
Araya - Burgess	-2.2549	1.000	1.18954643	1.000	2.60368656	0.968
Blanca - Burgess	0.30561670	1.000	1.91130983	1.000	-0.399	1.000
Araya - Desi	-5.9515	0.000	1.18954643	1.000	4.58494633	0.000
Blanca - Desi	-3.3909	0.073	1.91130983	1.000	1.58183622	1.000
Burgess - Desi	-3.6966	0.023	0.00000000	1.000	1.98125978	1.000
Araya - DJ	1.19230727	1.000	-1.9615	1.000	2.38068333	1.000
Blanca - DJ	3.75285248	0.018	-1.2398	1.000	-0.622	1.000
Burgess - DJ	3.44723578	0.059	-3.1511	0.171	-0.223	1.000
Desi - DJ	7.14379044	0.000	-3.1511	0.171	-2.204	1.000
Araya - Gannon	-2.4057	1.000	-0.2999	1.000	0.76118435	1.000
Blanca - Gannon	0.15481899	1.000	0.42186568	1.000	-2.242	1.000
Burgess - Gannon	-0.1508	1.000	-1.4894	1.000	-1.843	1.000
Desi - Gannon	3.54575695	0.041	-1.4894	1.000	-3.824	0.014
DJ - Gannon	-3.5980	0.034	1.66163422	1.000	-1.619	1.000
Araya - Iggy	-2.4459	1.000	0.08035537	1.000	0.64125817	1.000
Blanca - Iggy	0.11460626	1.000	0.80211877	1.000	-2.362	1.000
Burgess - Iggy	-0.1910	1.000	-1.1092	1.000	-1.962	1.000
Desi - Iggy	3.50554423	0.048	-1.1092	1.000	-3.944	0.008
DJ - Iggy	-3.6382	0.029	2.04188730	1.000	-1.739	1.000
Gannon - Iggy	-0.0402	1.000	0.38025308	1.000	-0.12	1.000
Araya - Jules	-3.9949	0.007	1.17336137	1.000	3.52075365	0.045
Blanca - Jules	-1.4692	1.000	1.88530440	1.000	0.55850408	1.000
Burgess - Jules	-1.7706	1.000	0.00000000	1.000	0.95249305	1.000
Desi - Jules	1.87563134	1.000	0.00000000	1.000	-1.002	1.000
DJ - Jules	-5.1710	0.000	3.10820456	0.198	1.17246207	1.000
Gannon - Jules	-1.6219	1.000	1.46917866	1.000	2.76992603	0.589
Iggy - Jules	-1.5822	1.000	1.09409933	1.000	2.88822049	0.407
Araya - Lou	-0.5389	1.000	-0.0201	1.000	-0.953	1.000
Blanca - Lou	2.02169471	1.000	0.70167455	1.000	-3.957	0.008
Burgess - Lou	1.71607800	1.000	-1.2096	1.000	-3.557	0.039
Desi - Lou	5.41263267	0.000	-1.2096	1.000	-5.538	0.000
DJ - Lou	-1.7312	1.000	1.94144309	1.000	-3.334	0.090
Gannon - Lou	1.86687572	1.000	0.27980887	1.000	-1.715	1.000
Iggy - Lou	1.90708844	1.000	-0.1004	1.000	-1.595	1.000
Jules - Lou	3.46335663	0.056	-1.1932	1.000	-4.461	0.001
Araya - Mario	-2.6922	0.745	-2.4666	1.000	2.84552117	0.466

Blanca - Mario	-0.1317	1.000	-1.7449	1.000	-0.158	1.000
Burgess - Mario	-0.4373	1.000	-3.6562	0.027	0.24183461	1.000
Desi - Mario	3.25924129	0.117	-3.6562	0.027	-1.739	1.000
DJ - Mario	-3.8845	0.011	-0.5051	1.000	0.46483784	1.000
Gannon - Mario	-0.2865	1.000	-2.1667	1.000	2.08433682	1.000
Iggy - Mario	-0.2463	1.000	-2.5470	1.000	2.20426300	1.000
Jules - Mario	1.33926446	1.000	-3.6064	0.033	-0.714	1.000
Lou - Mario	-2.1534	1.000	-2.4465	1.000	3.79898385	0.015
Araya - Monte	-1.7844	1.000	-2.8670	0.435	3.20926421	0.140
Blanca - Monte	0.77610558	1.000	-2.1452	1.000	0.20615409	1.000
Burgess - Monte	0.47048887	1.000	-4.0565	0.005	0.60557765	1.000
Desi - Monte	4.16704354	0.003	-4.0565	0.005	-1.376	1.000
DJ - Monte	-2.9767	0.306	-0.9054	1.000	0.82858088	1.000
Gannon - Monte	0.62128659	1.000	-2.5671	1.000	2.44807986	1.000
Iggy - Monte	0.66149931	1.000	-2.9473	0.337	2.56800604	1.000
Jules - Monte	2.23471508	1.000	-4.0013	0.007	-0.355	1.000
Lou - Monte	-1.2456	1.000	-2.8469	0.464	4.16272689	0.003
Mario - Monte	0.90780225	1.000	-0.4003	1.000	0.36374304	1.000
Araya - Nino	-3.4623	0.056	-1.3718	1.000	3.69888018	0.023
Blanca - Nino	-0.9018	1.000	-0.6500	1.000	0.69577007	1.000
Burgess - Nino	-1.2074	1.000	-2.5613	1.000	1.09519362	1.000
Desi - Nino	2.48916762	1.000	-2.5613	1.000	-0.886	1.000
DJ - Nino	-4.6546	0.000	0.58975101	1.000	1.31819685	1.000
Gannon - Nino	-1.0566	1.000	-1.0719	1.000	2.93769584	0.347
Iggy - Nino	-1.0164	1.000	-1.4521	1.000	3.05762202	0.234
Jules - Nino	0.57966848	1.000	-2.5265	1.000	0.12779929	1.000
Lou - Nino	-2.9235	0.363	-1.3517	1.000	4.65234287	0.000
Mario - Nino	-0.7701	1.000	1.09484189	1.000	0.85335901	1.000
Monte - Nino	-1.6779	1.000	1.49518382	1.000	0.48961597	1.000
Araya - PJ	-2.3132	1.000	-0.8107	1.000	2.88516619	0.411
Blanca - PJ	0.24730825	1.000	-0.0890	1.000	-0.118	1.000
Burgess - PJ	-0.0583	1.000	-2.0003	1.000	0.28147963	1.000
Desi - PJ	3.63824622	0.029	-2.0003	1.000	-1.7	1.000
DJ - PJ	-3.5055	0.048	1.15080367	1.000	0.50448285	1.000
Gannon - PJ	0.09248927	1.000	-0.5108	1.000	2.12398184	1.000
Iggy - PJ	0.13270199	1.000	-0.8911	1.000	2.24390802	1.000
Jules - PJ	1.71311262	1.000	-1.9731	1.000	-0.675	1.000
Lou - PJ	-1.7744	1.000	-0.7906	1.000	3.83862887	0.013
Mario - PJ	0.37900492	1.000	1.65589455	1.000	0.03964502	1.000
Monte - PJ	-0.5288	1.000	2.05623648	1.000	-0.324	1.000
Nino - PJ	1.14907859	1.000	0.56105266	1.000	-0.814	1.000
Araya - Smitty	-6.0580	0.000	1.17336137	1.000	4.17707605	0.003
Blanca - Smitty	-3.5323	0.043	1.88530440	1.000	1.21482648	1.000
Burgess - Smitty	-3.8337	0.013	0.00000000	1.000	1.60881545	1.000

Desi - Smitty	-0.1875	1.000	0.00000000	1.000	-0.345	1.000
DJ - Smitty	-7.2341	0.000	3.10820456	0.198	1.82878448	1.000
Gannon - Smitty	-3.6850	0.024	1.46917866	1.000	3.42624844	0.064
Iggy - Smitty	-3.6453	0.028	1.09409933	1.000	3.54454289	0.041
Jules - Smitty	-2.0358	1.000	0.00000000	1.000	0.64762901	1.000
Lou - Smitty	-5.5265	0.000	1.19317689	1.000	5.11756585	0.000
Mario - Smitty	-3.4024	0.070	3.60642314	0.033	1.37027126	1.000
Monte - Smitty	-4.2978	0.002	4.00131798	0.007	1.01147733	1.000
Nino - Smitty	-2.6428	0.863	2.52647775	1.000	0.52852311	1.000
PJ - Smitty	-3.7762	0.017	1.97305881	1.000	1.33116565	1.000
Araya - Tux	-1.9634	1.000	1.18954643	1.000	2.64234045	0.865
Blanca - Tux	0.59715895	1.000	1.91130983	1.000	-0.361	1.000
Burgess - Tux	0.29154225	1.000	0.00000000	1.000	0.03865389	1.000
Desi - Tux	3.98809692	0.007	0.00000000	1.000	-1.943	1.000
DJ - Tux	-3.1557	0.168	3.15107837	0.171	0.26165712	1.000
Gannon - Tux	0.44233997	1.000	1.48944415	1.000	1.88115611	1.000
Iggy - Tux	0.48255269	1.000	1.10919107	1.000	2.00108228	1.000
Jules - Tux	2.05820322	1.000	0.00000000	1.000	-0.914	1.000
Lou - Tux	-1.4245	1.000	1.20963528	1.000	3.59580314	0.034
Mario - Tux	0.72885562	1.000	3.65616926	0.027	-0.203	1.000
Monte - Tux	-0.1789	1.000	4.05651118	0.005	-0.567	1.000
Nino - Tux	1.49892929	1.000	2.56132736	1.000	-1.057	1.000
PJ - Tux	0.34985070	1.000	2.00027470	1.000	-0.243	1.000
Smitty - Tux	4.12130955	0.004	0.00000000	1.000	-1.571	1.000

Supplemental Table S2. Results of Dunn's post-hoc tests for proximity (Zoo Monitor data). Table includes Z scores and adjusted P values (significant differences highlighted in yellow). See Section 3.3.

Pair 1	Pair 2	Z score	P adj
BLUE	BROWN	-4.790	0.0000
BLUE	GREEN	-4.520	0.0001
BROWN	GREEN	0.276	1.0000
BLUE	PURPLE	-8.820	0.0000
BROWN	PURPLE	-3.986	0.0014
GREEN	PURPLE	-4.270	0.0004
BLUE	RED	-2.748	0.1259
BROWN	RED	2.006	0.9424
GREEN	RED	1.734	1.0000
PURPLE	RED	5.980	0.0000
BLUE	SIBLINGS	-11.857	0.0000
BROWN	SIBLINGS	-6.960	0.0000
GREEN	SIBLINGS	-7.252	0.0000
PURPLE	SIBLINGS	-2.952	0.0663
RED	SIBLINGS	-8.958	0.0000
BLUE	YELLOW	1.557	1.0000
BROWN	YELLOW	6.264	0.0000
GREEN	YELLOW	6.001	0.0000
PURPLE	YELLOW	10.239	0.0000
RED	YELLOW	4.247	0.0005
SIBLINGS	YELLOW	13.241	0.0000

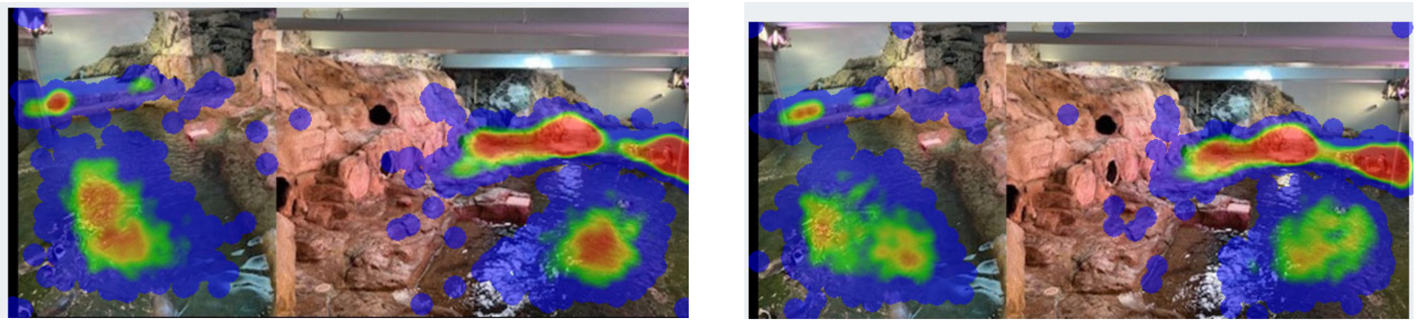

Figure S1. Blue pair
(a) Tux (male) (b) Burgess (female).

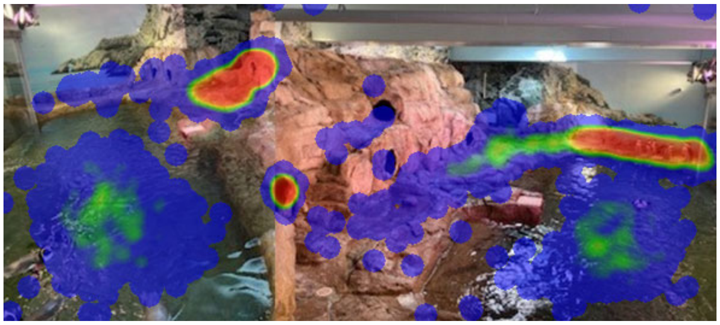

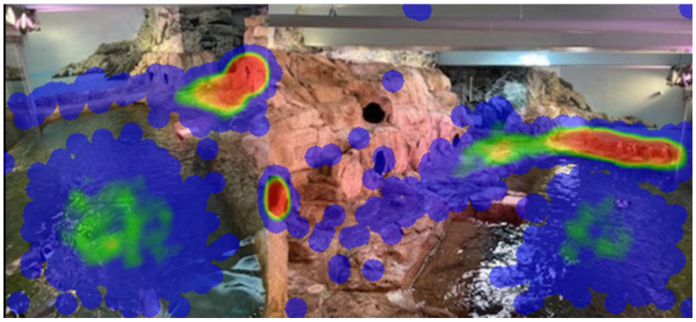

Figure S2. Red pair

(a) Mario (male)

(b) Monte (female).

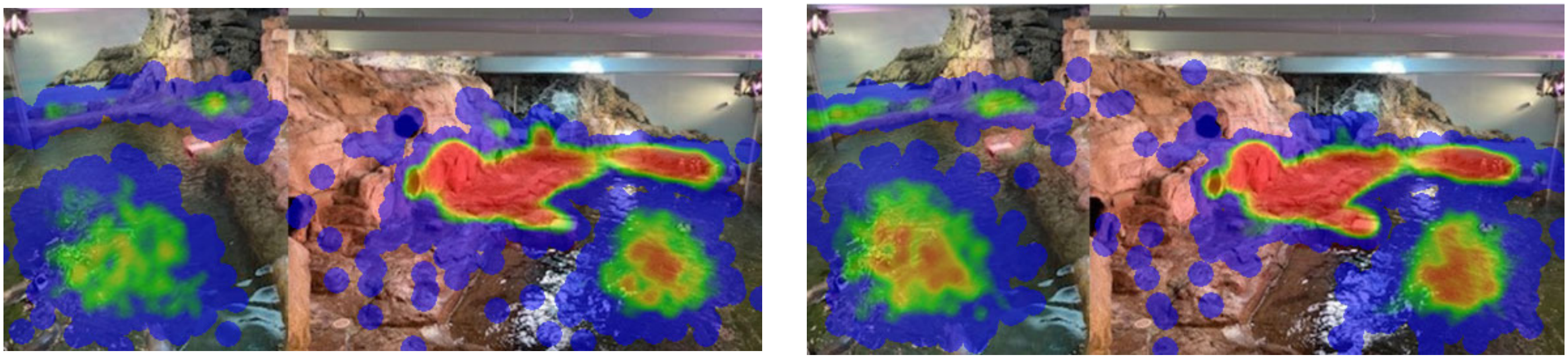

Figure S3. Green pair
(a) Iggy (male) (b) Gannon (female).

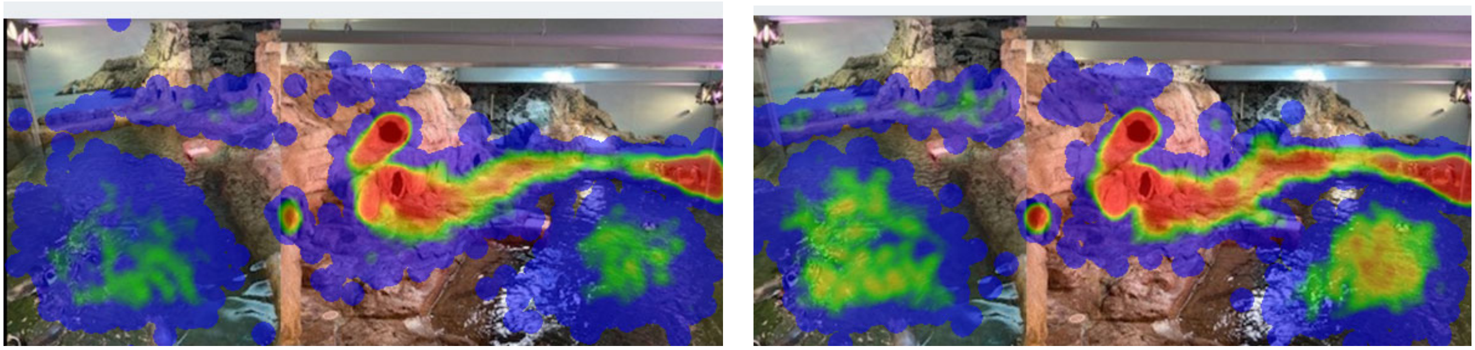

Figure S4. Brown pair (a) PJ (male) (b) Blanca (female).

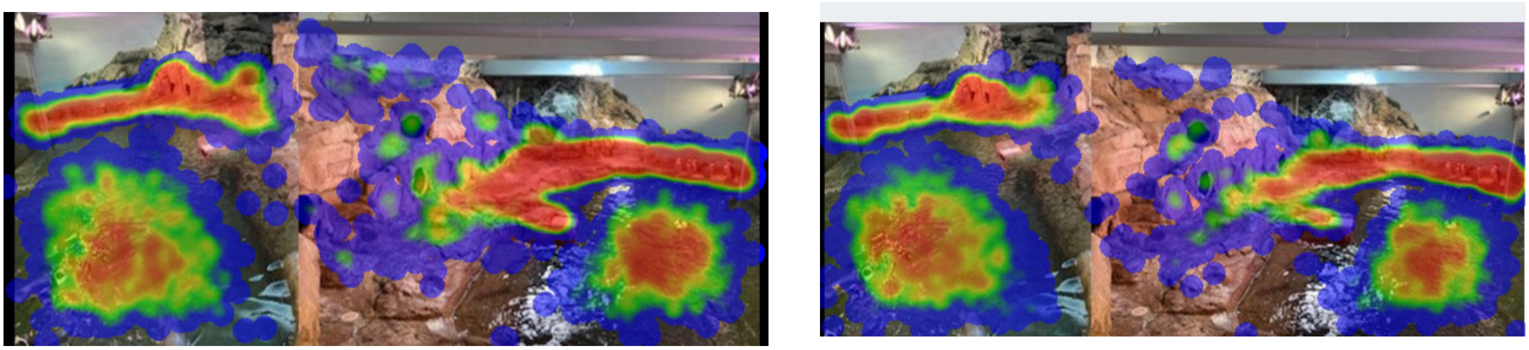

Figure S5. Siblings (a) Smitty (male)   (b) Jules (female).

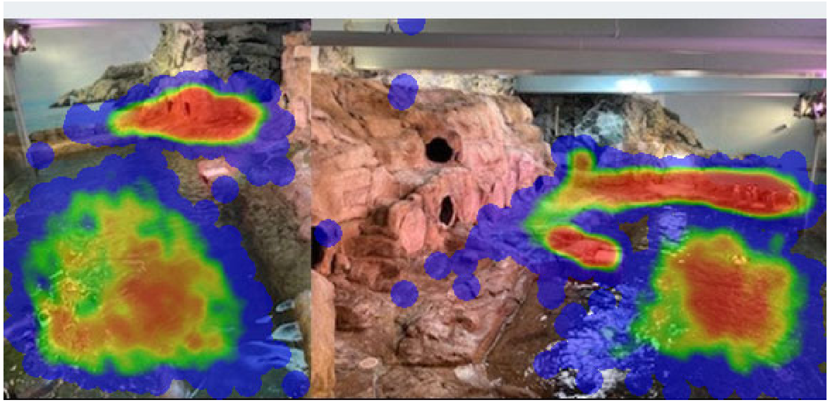

Figure S6. Single penguin Desi (male), unpaired.
